# Neural Mechanisms Underlying Paradoxical Performance for Monetary Incentives Are Driven by Loss Aversion

**DOI:** 10.1016/j.neuron.2012.02.038

**Published:** 2012-05-10

**Authors:** Vikram S. Chib, Benedetto De Martino, Shinsuke Shimojo, John P. O'Doherty

**Affiliations:** 1 Division of Biology, California Institute of Technology, Pasadena, CA 91125, USA; 2 Computation and Neural Systems, California Institute of Technology, Pasadena, CA 91125, USA; 3 Division of Humanities and Social Sciences, California Institute of Technology, Pasadena, CA 91125, USA; 4 Psychology and Language Sciences, University College of London, London WC1H 0AP, UK

## Abstract

Employers often make payment contingent on performance in order to motivate workers. We used fMRI with a novel incentivized skill task to examine the neural processes underlying behavioral responses to performance-based pay. We found that individuals' performance increased with increasing incentives; however, very high incentive levels led to the paradoxical consequence of worse performance. Between initial incentive presentation and task execution, striatal activity rapidly switched between activation and deactivation in response to increasing incentives. Critically, decrements in performance and striatal deactivations were directly predicted by an independent measure of behavioral loss aversion. These results suggest that incentives associated with successful task performance are initially encoded as a potential gain; however, when actually performing a task, individuals encode the potential loss that would arise from failure.

## Highlights

► Very high monetary incentives result in poor behavioral performance ► During incentive presentation, striatal activity increases with increasing incentives ► During task performance, striatal activity decreases with increasing incentives ► Performance and striatal responses are predicted by behavioral loss aversion

## Teaser

Chib et al. find that very large financial incentives can lead to the paradoxical response of poor work performance. Imaging results suggest that these decreases in performance are caused by individuals encoding the potential monetary loss that would arise from failure.

## Introduction

It is generally assumed that an increase in financial incentive provided for work will result in greater performance (Lazear, 2000). The reasoning behind this idea is that larger incentives increase a worker's motivation, which, in turn, elicits improved behavioral output and performance. However, recent behavioral experiments suggest a more idiosyncratic interplay between incentives and performance (Ariely et al., 2009): when executing skilled tasks, individuals' performance increases as the level of incentive increases only up to a point, after which greater incentives become detrimental to performance. Despite the ubiquity of performance-based incentive schemes in the workforce, the neural and psychological underpinnings of the relationship between incentives and performance are not well understood.

Although the relationship between financial incentives and performance has received limited investigation, the paradoxical relationship between arousal and performance has long been reported in the psychological literature (Baumeister, 1984; Martens and Landers, 1970; Wood and Hokanson, 1965; Yerkes and Dodson, 1908). Keeping in mind that arousal is closely associated with motivation, behavioral economics has borrowed theories from psychology to explain incentive based decrements (Ariely et al., 2009; Camerer et al., 2005).

These psychological theories attempt to provide explanations as to why external stressors such as presence of an audience or social stereotypes might have detrimental effects on behavioral performance—commonly termed “choking under pressure” (Baumeister, 1984; Beilock et al., 2004). A number of theories have been proposed to account for the choking phenomenon, including distraction theories and explicit monitoring theories. Distraction theories propose that pressure creates a distracting environment that shifts attentional focus to task-irrelevant cues, such as worries about the situation and its consequences (Beilock and Carr, 2001; Lewis and Linder, 1997; Wine, 1971). In contrast, explicit monitoring theories suggest that the presence of a stressor acts to wrest control of behavior from a habit-based instrumental system involved in the implementation of skilled motor acts, to a more goal-directed instrumental system in which actions must be selected in a deliberative manner (requiring on-going monitoring of performance) (Baumeister, 1984; Beilock and Carr, 2001; Beilock et al., 2004; Langer and Imber, 1979).

At the neural level, very little is known about the mechanisms underpinning performance decrements in stressful environments. Mobbs et al. (2009) found that the degree of subjects' midbrain activation during a challenging task was correlated with their performance decrement for large incentives. They interpreted this neural response as an “over-motivation” signal for the high rewards associated with successful task performance.

Another region known to play an important role in mediating interactions between rewards and motor performance is the ventral striatum. A number of studies have found this region to be involved in mediating the effects of rewards on increases in motor performance (Kurniawan et al., 2010; Pessiglione et al., 2007; Schmidt et al., 2008). The ventral striatum has been implicated in interactions between a Pavlovian system in which reflexive conditioned responses come to be elicited by a stimulus that predicts the subsequent delivery of a reward, and an instrumental system in which actions are selected flexibly in order to increase the probability of obtaining reward (Bray et al., 2008; Dickinson and Balleine, 1994; Talmi et al., 2008). In Pavlovian to instrumental transfer, instrumental responding for reward can be enhanced as a result of the presence of a reward predicting Pavlovian stimulus, an effect that is abolished in rodents following lesions of the ventral striatum (Corbit and Balleine, 2005). Furthermore, fMRI studies of humans have revealed activity in the ventral striatum during Pavlovian-to-instrumental transfer (Bray et al., 2008; Talmi et al., 2008). All of the above studies have focused on the role of ventral striatum in mediating enhancements in responding, as opposed to decrements. In contrast, in this study we aimed to investigate the role of the ventral striatum in mediating response decrements as a function of large incentives.

**Figure 1:**
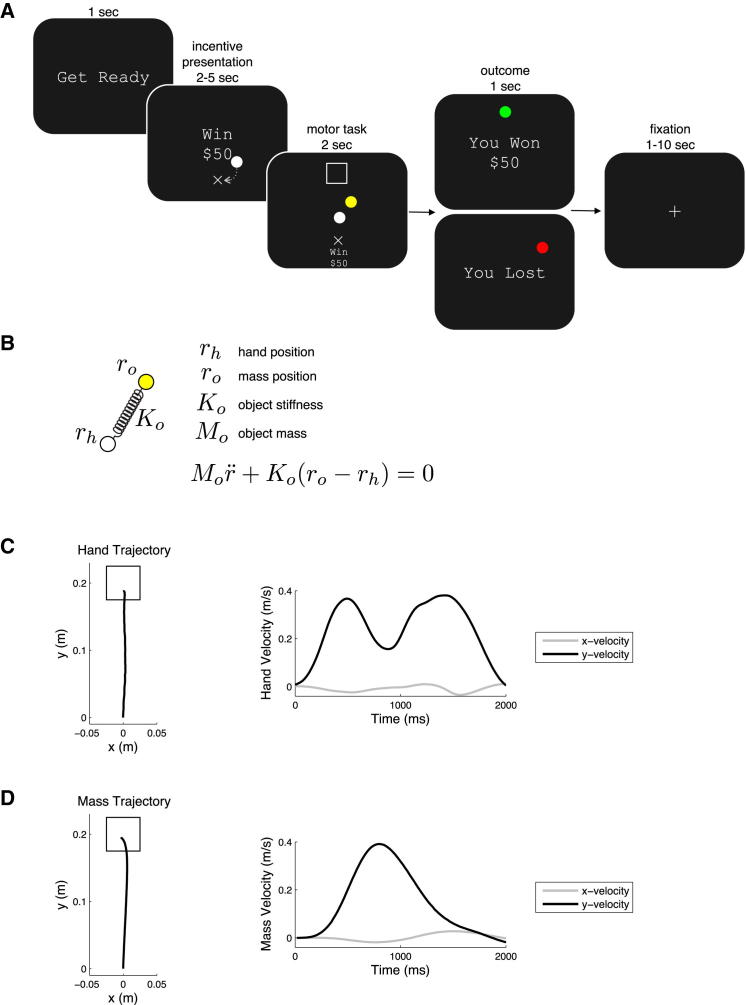
The Incentive-Based Motor Task (A) At the beginning of each trial participants were presented with the incentive (e.g., Win $50) for which they were playing. During incentive presentation, to initiate the motor task, participants placed their white hand cursor in the start position (X) for a random amount of time (2–5 s). During the task, a target (□) appeared that was registered to a position 20 cm distal from the start. To successfully achieve the task, participants had to place their hand cursor and a mass cursor into the target within 2 s, while achieving a final velocity below 0.02 m/s. At the end of the trial they were shown a message indicating the outcome of their performance. In the case that a participant successfully placed the spring-mass in the target, a positive message was displayed (”You Won $50”); otherwise, the participant was informed of her negative outcome (“You Lost”). (B) The spring-mass task provided us with a well defined dynamic system. The control input of the system was (r_h_) the position of the hand. Thus movements of the hand resulted in oscillations of the mass cursor. These equations assumed a zero rest length of the spring. We defined *M*_0_ = 3 kg and *K*_0_ = 120 Nm^−1^. The state equations for the system were integrated in real-time to compute the instantaneous position of the object for each corresponding position of the hand. Reaching movements were always initiated with the hand and object both at rest at the same position. As the participant began to move her hand the mass cursor would begin to oscillate. Because the dynamics of the task were completely novel to participants they had to learn what hand movements would result in controlled movements of the mass cursor. (C and D) After training participants executed bimodal hand velocity profiles (C) to achieve smooth bell-shaped velocity profiles of the mass cursor (D). This allowed the participant to direct both the hand and mass cursors into the target.

To this end, we used a novel motor control paradigm in conjunction with functional magnetic resonance imaging (fMRI). Participants performed the highly-skilled motor task of controlling a virtual spring-mass system (Figure 1B). This dynamic system was chosen because it was completely novel to participants, and thus allowed us to evaluate performance uncorrupted by participants' previous experiences or expertise (Dingwell et al., 2002). During trials participants moved both their hand and the mass from a start position to a target 20 cm away. A successful trial consisted of both the hand and mass being placed in the target, subject to velocity constraints.

The experiment took place on two consecutive days. On the first day of the experiment, participants trained on 500 repeated trials with the spring-mass system. After training, we determined participants' rates of success at various target sizes. This thresholding allowed us to tailor standard difficulty levels for each participant. On the second day, participants performed the testing phase and were scanned with fMRI while they controlled the spring-mass system with the purpose of obtaining reward. While in the magnet, on Day 2 of the experiment, participants performed trials for a range of incentives (i.e., $0, $5, $25, $50, $75, $100) and at two difficulty levels (easy and hard).

## Results

### Behavioral and Neural Responses

**Figure 2:**
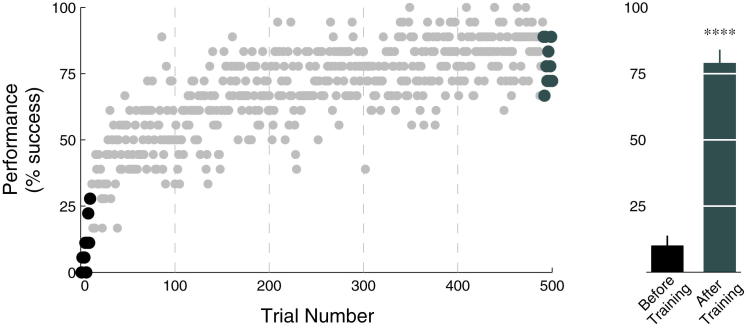
Behavioral Performance during Training Training performance represented as the percentage of successful trials computed across participants, for each trial number. Gray dashed lines indicate the beginning of each session of training. The bar graph represents the group mean of the first and last 10 trials of training. A significant increase in performance was seen after training (^∗∗∗^^∗^p < 0.0001).

**Figure 3:**
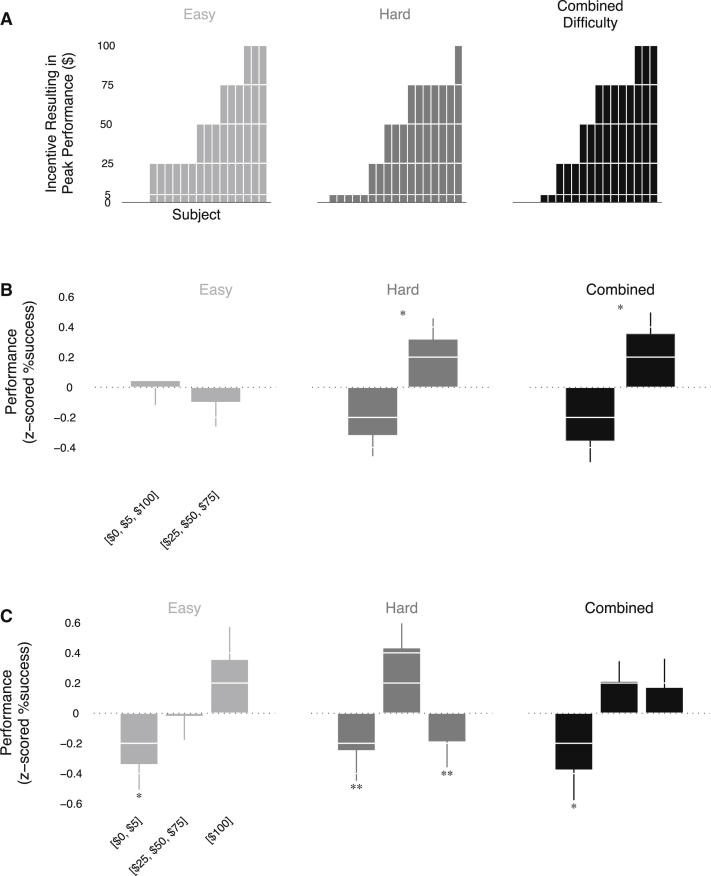
Behavioral Performance during Scanning (A) Participants exhibited individual differences in the incentive level at which their performance peaked. (B) At the hard and combined difficulty levels, and not the easy level, we found that participants had greater performance in the middle range of incentive as compared to the extremes of incentive. (C) Dividing incentive levels, at the hard difficulty level, we found that performance improved with increasing incentives up to a point. Further increases in incentives significantly decreased performance relative to peak performance. Planned comparisons of performance, relative to performance in the middle range of incentives, found significant decrements at the extremes of incentive for the hard condition (^∗^p < 0.05; ^∗∗^p < 0.01; ^∗∗∗^p < 0.001). Error bars represent SEM. See also Figure S2.

Behavioral results from the training phase (Day 1) indicated that control of the spring-mass system was initially challenging for participants, but after repeated practice they were able to increase their performance (Figure 2). In the subsequent experiment, we found that participants exhibited peak performance over the range of incentive levels and the bulk of participants reached peak performance at an incentive level less than $100 (Figure 3A). This variability in performance responses for incentives was likely due to participants' differences in subjective value for incentives (Ariely et al., 2009). To account for differences in behavioral performance variance between participants, each participants' measures of performance were separately standardized (Z-scored) across incentive categories. We computed group statistics on behavioral responses to incentive using these standardized performance measures.

To examine participants' behavioral responses to incentive, we compared performance at the extremes of incentive with performance in the middle range of incentives (see the Data Analysis section for details). At the hard (t(17) = 2.20, p = 0.04) and combined (t(17) = 2.47, p = 0.02) difficulty levels, and not the easy level (t(17) = 0.42, p = 0.70), we found that participants had greater performance in the middle range of incentive as compared to the extremes of incentive (Figure 3B). We also found a significant interaction between these incentive categories and difficulty (F[1,68] = 6.30, p = 0.01). Further dividing incentive levels (Figure 3C), we found significant main effects of incentive on performance in the hard condition (F[2,51] = 5.07, p = 0.01), and not the easy (F[2,51] = 2.27, p = 0.11) or combined (F[2,51] = 2.10, p = 0.13) conditions. We again found a significant interaction between incentive categories and difficulty (F[2,102] = 3.60, p = 0.03). In the hard level we found that participants' performance improved with increasing incentive level up to a point; beyond this point, further increasing incentives significantly decreased performance relative to peak performance (Figure 3C).

**Figure 4:**
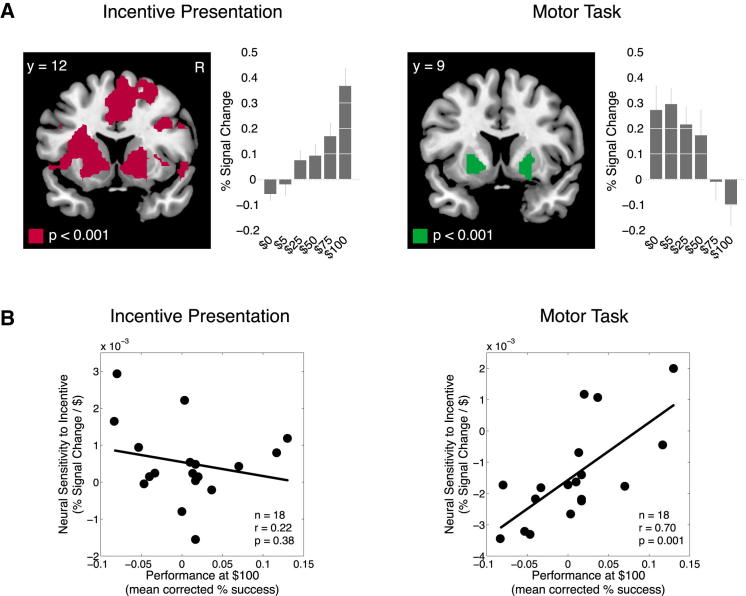
fMRI Results (A) Activity in ventral striatum was positively correlated with incentive level at the time of incentive presentation (x = 12; y = 12; z = −6), and negatively correlated with incentive level at the time of the motor task (x = 21; y = 9; z = −9). All contrasts are significant at p < 0.05, small volume corrected. (B) Plots of the correlations between neural sensitivity and mean corrected performance at the $100 incentive level (for the combined difficulty) for each participant. Error bars denote SEM. Participants' performance was mean corrected to adjust for differences in overall performance between participants. See also Figure S1 and Tables S1, S2, and S3.

Because participants performed this task in the fMRI scanner, we were able to examine the underlying brain activity involved in generating their performance responses. Figure 4A shows that, at the time of incentive presentation, the blood oxygen level-dependent (BOLD) signal in ventral striatum increased with the magnitude of incentive (cluster sizes > 100 voxels; right cluster peak: [x = 12; y = 12; Z = −6], T = 6.51; left cluster peak: [x = −21; y = 15; Z = −3], T = 5.59). Conversely, we found that striatal activation during the motor task decreased with respect to the magnitude of incentive (cluster sizes > 100 voxels; right cluster peak: [x = 21; y = 9; Z = −9], T = 4.15; left cluster peak: [x = −18; y = 6; z = −6], Z = 3.89). These results point to a rapid switching, in the direction of striatal activity, between the presentation of incentive and subsequent performance of the motor action. We performed a simulation of our fMRI design to confirm that the striatal deactivation response was not due to the BOLD response leaking from the incentive presentation phase into the motor task phase (Figures S1A and S1B available online). We also performed an analysis of our data to confirm that the striatal deactivation was not a physiological artifact (Figure S1C).

Strikingly, the only brain region commonly active between the time of incentive presentation (Table S1) and the execution of the motor task (Table S2) was bilaterally encompassing ventral striatum (Table S3). Furthermore, additional whole brain analyses did not reveal any brain regions that were directly correlated with participants' parabolic behavioral performance or interactions between incentive level and task difficulty (see Supplemental Information for details, Figure S1E). These analyses provided us with further evidence of the ventral striatum's integral role in mediating participants' responses during performance for incentives.

The idiosyncratic pattern of striatal activity we observed (i.e., activation at the time of incentive presentation and deactivation at the time of action) resembles that reported for participants experiencing potential monetary gains and losses (Tom et al., 2007; Yacubian et al., 2006). Tom et al. (2007) found that ventral striatum was activated by the prospect of gains, and deactivated by the prospect of losses, and that such deactivation was strongly correlated with a behavioral measure of loss aversion.

The findings of Tom et al. (2007), in conjunction with our results, led us to develop a new hypothesis regarding the role of ventral striatum in mediating performance decrements for large incentives: deactivation of ventral striatum during motor action reflects evaluation of the potential loss (of a presumed gain) that would arise from failure to successfully achieve the task. Essentially, larger incentives are framed as larger potential losses, and as these perceived potential losses increase (in the highest incentive conditions) they are manifested as performance decrements. Because this hypothesis is generated in part from a “reverse-inference” (Poldrack, 2006), we needed to obtain additional evidence in order to provide direct empirical support. Our hypothesis led to the following predictions: (1) striatal deactivation at the time of motor action would predict the extent of individuals' decrements in behavioral performance; (2) activity in ventral striatum during motor action would relate to an individual's behavioral loss aversion (i.e., the more loss averse a participant, the greater her ventral striatal deactivation during motor action); and (3) a participant's degree of behavioral loss aversion would be predictive of her propensity to exhibit performance decrements for large incentives, as well as the level of incentive that resulted in peak performance.

### Loss Aversion Predicts Neural Responses to Incentives

To test the first prediction, we examined the extent to which a participant's decrease in performance at the highest incentive level was related to her neural sensitivity to incentive. For this analysis we performed correlations between participants' behavioral performance at the $100 incentive level and activity in the striatum. Neural sensitivity to incentive was defined as the slope of the relationship between BOLD percent signal change and incentive level; a positive neural sensitivity corresponded to neural activation, whereas a negative activity was indicative of deactivation.

In keeping with the first prediction, we found significant correlations between levels of striatal deactivation at the time of the motor task and performance decrements at the $100 incentive level (Figure 4B; *r* = 0.70; p = 0.001). Critically, no significant relationship between neural sensitivity and performance was found at the time of incentive presentation (*r* = 0.22; p = 0.38). Using a cross-product term in a multiple regression model, we also found a significant interaction between neural sensitivity during incentive presentation and the motor task and performance (statistics for interaction term: t(14) = 4.18; p = 0.001).

**Figure 5:**
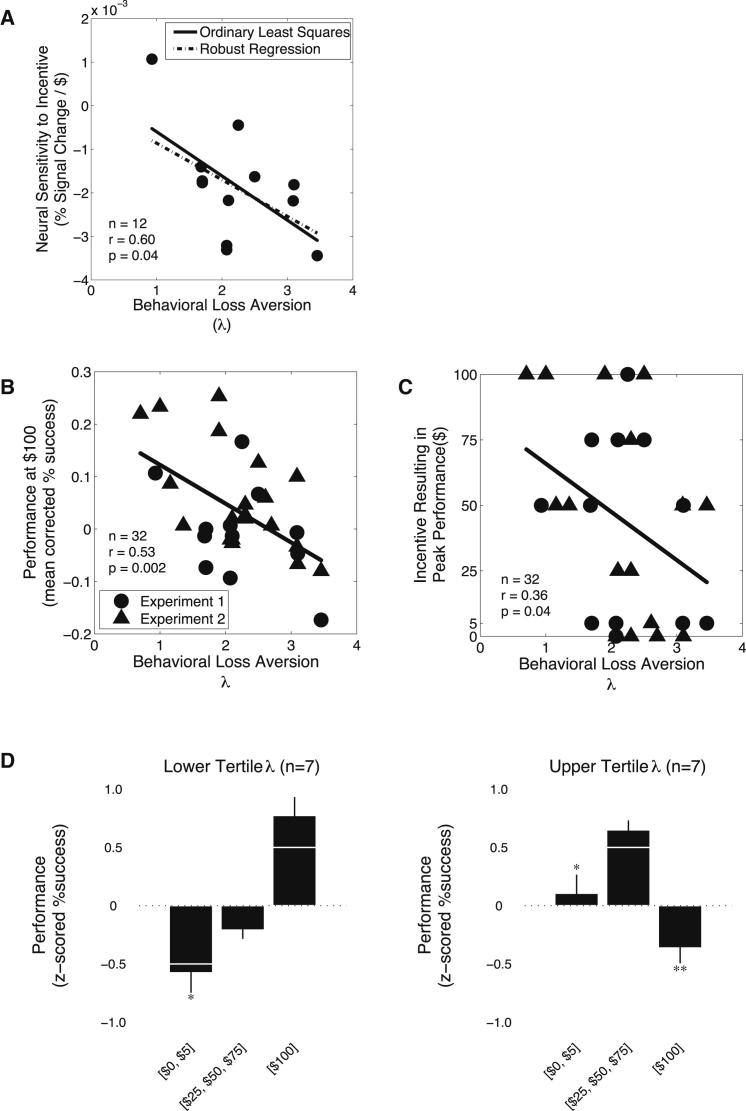
Loss Aversion across Participants (A) The correlation between behavioral loss aversion for each participant and their neural sensitivity to incentive (at the time of the motor task). A robust regression revealed that a candidate outlier did not have an impact on the correlation between neural sensitivity and loss aversion. This candidate outlier was not in fact an outlier as it was within 2 SD of the mean (λ = 0.94; group mean ± 2^∗^SD, λ = 2.22 ± 1.44). We also performed an additional fMRI study that duplicated the relationship between neural sensitivity and loss aversion. See also Figure S3. (B and C) Plots of the correlations between (B) behavioral loss aversion and mean corrected behavioral performance at the $100 incentive level (for the hard difficulty level) (C), and incentive resulting in peak performance (for the hard difficulty level) (C). Plots (B) and (C) contain data from the initial (n = 12) and the follow-up (n = 20) experiments. (D) Behavioral performance from the follow-up experiment (n = 20, for the hard difficulty level). For this analysis we separated participants into tertiles based on the extent of their loss aversion. We found a main effect of incentive in the lower (F[2,18] = 5.38, p = 0.02) and upper (F[2,18] = 4.29, p = 0.04) tertile of loss aversion. Planned comparisons of performance, relative to performance in the middle range of incentives, found significant decrements (^∗^p < 0.05; ^∗∗^p < 0.01) at the extremes of incentive for participants in the upper tertile and not in the lower tertile. Error bars represent SEM.

To test the second prediction we recalled a subset of participants (n = 12) who originally participated in these experiments and tested them on a behavioral loss aversion task. This task was the same as that used by Tom et al. (2007), and allowed us to determine a measure λ, indicating how heavily participants weighed losses compared to gains. This subset of participants was found to have a median λ estimate of 2.09 (interquartile range [IQR] 1.09). These values of λ are similar to those reported in previous studies (Bateman et al., 2005; Gachter et al., 2007; Tom et al., 2007; Tverskey and Kahneman, 1992). We found significant correlations between increasing behavioral loss aversion and striatal deactivation during motor action (Figure 5A; *r* = 0.60; p = 0.04; Figure S3). Importantly, we did not find a significant correlation between neural sensitivity during incentive presentation and participants' behavioral loss aversion (*r* = 0.30; p = 0.34). We also found a significant interaction between neural sensitivity during incentive presentation and the motor task and loss aversion (statistics for interaction term: t(8) = 2.40 p = 0.05). These results illustrate that differences in behavioral loss aversion were indicative of neural responses during motor action.

### Loss Aversion Predicts Behavioral Responses to Incentives

To test the third prediction, and to reach an adequate sample size to test behavioral correlations, we included an additional 20 participants who performed the motor task, the behavioral loss aversion task, and a risk aversion task outside the fMRI scanner. A group comprised of both the subset of imaging participants (n = 12), and the additional participants (n = 20) had a median λ estimate of 2.10 (IQR 0.85). We found a highly significant (*r* = 0.53; p = 0.002) relationship between increasing behavioral loss aversion and the proclivity to show performance decrements in the hard difficulty level (Figure 5B), but not in the easy difficulty level (*r* = 0.22; p = 0.23). We also found a significant relationship (*r* = 0.36; p = 0.04) between decreasing behavioral loss aversion and the level of incentive resulting in peak behavioral performance in the hard difficulty level (Figure 5C), but not in the easy difficulty level (*r* = 0.24; p = 0.19). Those participants with greater behavioral loss aversion exhibited peak performance at lower incentive levels and more impaired performance for high incentives. The additional group of participants (n = 20) exhibited a wide range of λ's and separating these participants based on the degree of their loss aversion, we found that those that were less loss averse followed a monotonic response to incentives, whereas more loss averse participants exhibited the paradoxical response to incentives (Figure 5D). These results provide evidence that participants frame their performance for incentives, during highly skilled tasks, in terms of the loss of a presumed gain that would arise from failure. Moreover, this encoding of loss aversion drives participants' behavioral performance for incentive.

Loss aversion represents a tendency to value losses greater than equal magnitude gains. Risk aversion, on the other hand, is a more general aversion to increased variance in potential gains or losses. To ensure a loss aversion-based hypothesis and not a general aversion to risk was responsible for our findings, we had participants in the follow-up experiment (n = 20) perform another decision-making task in which they made choices regarding risky gambles that did not include potential losses. Using participants' responses from this task we were able to calculate a measure α that represented their risk aversion. Participants had a median α estimate of 0.83 (IQR 0.20), indicating that they were on average risk averse. Importantly, no significant correlations were found between our behavioral measures of performance and risk aversion (Table 1). This provides further evidence that an individual's incentive resulting in peak performance and her performance decrements for large incentives are due specifically to loss aversion.

### A Prediction Error Model Does Not Describe Neural Responses to Incentives

Given that the striatum is also known to encode signals resembling a rewarded prediction error (McClure et al., 2003; O'Doherty et al., 2003; Pagnoni et al., 2002), we performed a simulation to determine if the deactivations observed during the motor task could be elicited as a byproduct of prediction error signaling. For this analysis we considered a temporal difference (TD) model of prediction error (PE), where a prediction error δ was generated from a difference between a predicted value *V*(*t*) at time *t* and a predicted value *V*(*t* + 1) at time *t* + 1 (Sutton and Barto, 1990):

δ=V(t+1)−V(t).

In our experiment, participants trained the day before the rewarded portion of the experiment and thus generated an expectation of their probability of success given a presented target size, and an average probability of success over all trials. We will assume that participants had, through training, learned the probability of success on easy trials *P_easy_* = 0.80, and on hard trials *P_hard_* = 0.60. Therefore, on average *P_combined_* = 0.70. With these probabilities of success we can generate the PE signals that would occur through the course of a trial and examine if these PEs match our neural data.

**Figure 6:**
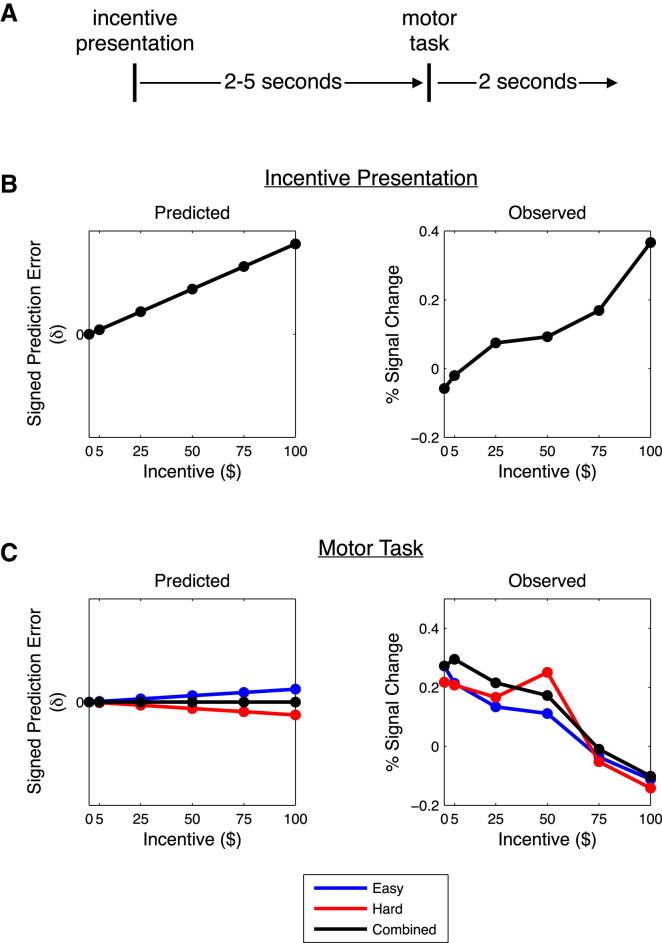
A Prediction Error Model Does Not Describe Neural Responses to Incentives (A) We simulated prediction error (PE) signals at the time of incentive presentation and the time of motor task execution. (B) At the time of incentive presentation the PE model is in correspondence with observed striatal activations. (C) At the time of the motor task the PE model predicts positive prediction errors for easy trials, negative predictions errors for hard trials, and no prediction errors for combined difficulty. We observe striatal deactivations in easy, hard, and combined trials. Observed percent signal change data were extracted from average voxel activity in a ventral striatal ROI in contrasts for easy, hard, and combined difficulty conditions. See also Figure S4.

At the beginning of a trial the predicted reward *V*(*t*_0_) is zero for each time *t* until the time of incentive presentation *t_presentation_*. The initial presentation of incentive results in a positive prediction error δ = *P_combined_*^∗^*V*(*t_presentation_*) − 0. At *t_presentation_* participants are not given any cues regarding trial difficulty, therefore their probability of success is *P_combined_*. These expectations result in positive prediction errors that increase with the magnitude of the incentive offered (Figure 6B). It can be seen that this PE response mirrors the striatal activations we observed during incentive presentation.

When the motor task begins at *t_motor_*, participants update their prediction error depending on the difficulty of the trial: easy trials δ = *P_easy_*^∗^*V*(*t_motor_*) − *P_combined_*^∗^*V*(*t_presentation_*); hard trials δ = *P_hard_*^∗^*V*(*t_motor_*) − *P_combined_*^∗^*V*(*t_presentation_*). This results in different PE responses for the different trial difficulties (Figure 6C). Easy trials result in positive PEs that scale with the magnitude of the incentive, whereas hard trials result in negative PEs that also scale with the magnitude of incentive.

Predicted PE responses for hard trials mimic our observed responses in striatum, however striatal responses for easy and combined trials do not align with the predictions of the PE model. Instead, we see that observed responses for easy trials are exactly opposite those of the PE model (Figure S4). Furthermore, observed responses for the combined trials show deactivation, whereas the model predicts no PE response. Overall, the results of our simulation illustrate that a TD PE model is not sufficient to describe our observed neural responses to incentives.

One might also consider a modified version of the PE model that incorporates a loss aversion parameter such that negative prediction errors loom larger than positive prediction errors. However, such a revised PE model still does not capture the pattern of deactivations observed in the easy condition of our current task.

### Brain Regions Showing an Interaction between Task Performance and Incentive

**Figure 7:**
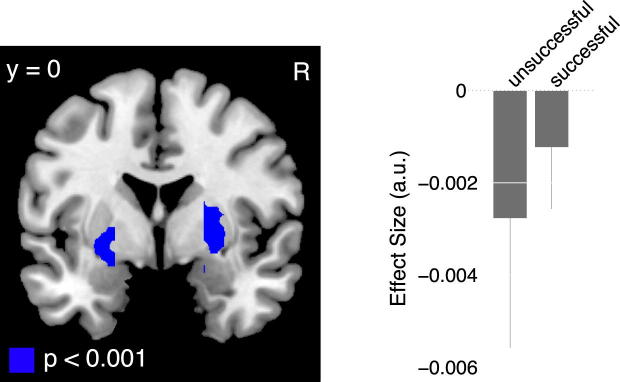
Interaction between Task Performance and Incentive Level This contrast illustrates brain regions, at the time of the motor task, that showed a greater deactivation as a function of incentive level on unsuccessful compared to successful trials (contrast: [unsuccessful ($0, $5, $25, $50, $100)] − [successful ($0, $5, $25, $50, $100)]. Ventral striatum [x = 27; y = 0; z = 0], significant at p < 0.05, small volume corrected. Error bars represent SEM. See also Figure S5 and Table S4.

To examine differences in brain activity as a function of unsuccessful versus successful performance, we contrasted unsuccessful and successful trials at the time of the motor task. We also examined an interaction between performance (i.e., unsuccessful and successful trials) and incentive level. We found no significant main effect of task performance. However, we did find a significant interaction between performance and incentive in the ventral striatum (Figure 7; Table S4), such that this region showed a greater deactivation as a function of incentive during unsuccessful trials compared to successful trials (cluster sizes > 100 voxels; right cluster peak: [x = 27; y = 0; Z = 0], T = 6.96; left cluster peak: [x = −27; y = 3; Z = −3], T = 5.05). This region overlapped with the portion of the ventral striatum we found to be positively correlated with incentive at the time of incentive presentation and negatively correlated with incentive during the motor task (Figure S5). No other brain region showed a significant effect in this contrast (Table S4).

The finding of a similar pattern of deactivation in the striatum during unsuccessful and successful trials suggests that on all trials participants evaluate the prospect of losing. This loss aversion is manifested irrespective of participants' confidence about the likelihood of success as their motor execution progresses on successful trials, and irrespective of the eventual outcome of a particular trial.

## Discussion

Our results provide insights into the potential contribution of the ventral striatum in mediating the interaction between incentives and behavioral performance. At the time of incentive presentation increased incentives result in striatal activation. This striatal activation is consistent with a wealth of evidence showing that the striatum encodes a motivational signal associated with the size of a potential reward (Breiter et al., 2001; Elliott et al., 2003; Pessiglione et al., 2007; Tom et al., 2007). However, we find that during task execution the same portion of striatum deactivates in manner that is indicative of loss aversion and eventual performance decrements. It is also important to note that these findings are not confounded by differences in behavioral performance between conditions, because the reported fMRI results are based on trials in which the motor act was ultimately successfully performed. Furthermore, a careful analysis of participants' movement trajectories yielded no significant differences in a variety of kinematic measures as a function of incentive level on successful trials (Figure S2). This indicates that basic differences in the pattern of elicited motor behavior cannot explain the observed fMRI results.

A recent imaging study found decreases in behavioral performance and increases in midbrain activity in response to a large incentive (Mobbs et al., 2009). The authors interpreted this response as an “over motivation” signal for the high reward associated with successful task performance. Here we show that “arousal” or overmotivation is unlikely to be a complete account for such performance decrements. The increasing positive responses we observed in striatum (that could be related to arousal [Cooper and Knutson, 2008]), at the time of incentive presentation, were not correlated with performance decrements. Instead, only the decreasing activity observed during actual motor action correlated with these decrements in performance. Furthermore, loss aversion and not other arousal provoking behavioral tendencies, such as risk-aversion (Lo and Repin, 2002), were found to be correlated with performance decrements and striatal deactivation during motor action. Although the Mobbs et al. (2009) study did not implicate ventral striatum in the choking effect, instead identifying midbrain and dorsal striatum, it is important to note that their study differed from ours in the manner in which incentives were delivered. In our study actual monetary rewards were only delivered at the end of the experiment, whereas in the Mobbs et al. (2009) study, incentives were accrued after every trial. Such differences in experimental design could potentially account for the different pattern of results.

One plausible mechanistic account of our findings relates to a long hypothesized role for the ventral striatum as a limbic-motor interface-mediating interactions between systems for Pavlovian valuation and instrumental responding (Alexander et al., 1990; Balleine, 2005; Cardinal et al., 2002; Mogenson et al., 1980). Whereas previous literature has focused on the role of the ventral striatum in mediating the effect of reward-predicting cues in increasing or enhancing instrumental performance for reward, our findings also point to a potential contribution of this region in performance decrements. In our experiment it is likely that, during motor performance, the prospect of losing elicits participants' aversive Pavlovian conditioned responses (Dayan and Seymour, 2008). These aversive responses could include motor withdrawal and avoidance, as well as engagement of attention or orienting mechanisms away from the task. At the level of motor execution, competing aversive Pavlovian responses could interfere with the motor commands necessary for successful execution of skilled instrumental responses.

The main output pathway of the ventral striatum is via the ventral pallidum (Graybiel, 2000; Grillner et al., 2005; Groenewegen, 2003). The ventral pallidum projects to the thalamus, which, in turn, sends motor signals to cortical areas (Graybiel, 2000; Grillner et al., 2005; Groenewegen, 2003). The ventral striatum also sends direct projections to brainstem areas such as the pedunculopontine nucleus, which is implicated in voluntary motor control (Lavoie and Parent, 1994; Mena-Segovia et al., 2004; Semba and Fibiger, 1992). Accordingly, it is possible that interference of the motor system from a ventral striatal motivation signal could occur either at the level of the cortex or the brainstem. Considerable further work will be needed to establish how ventral striatal signals come to act on the motor system, both in the domains of performance increments and performance decrements.

Our findings also have implications for other psychological explanations of choking effects. As noted above, according to the loss aversion theory, participants will likely engage mechanisms associated with being in an aversive state. This could include allocation of attentional resources away from the task. In this sense divergence of attention may provide a potential role in modulating performance. However, we did not find evidence of behavioral decrements correlating with the fronto-parietal attention network (Corbetta and Shulman, 2002), as might be predicted by attentional theories. Although this does not completely rule out the role of attention in the phenomenon, such effects (if present) appear not to be mediated by brain systems typically implicated in controlling attention.

Explicit monitoring theories suggest that performance decrements can be caused by the transfer of behavioral control from an automatized habit system to a goal-directed deliberative system (Baumeister, 1984; Beilock and Carr, 2001; Beilock et al., 2004; Langer and Imber, 1979). Considerable progress has been made in identifying brain systems involved in goal-directed and habitual control, with the ventromedial prefrontal cortex and anterior dorsal striatum implicated in the former, and the posterolateral striatum implicated in the latter (Balleine and Dickinson, 1998; Balleine and O'Doherty, 2010; Corbit and Balleine, 2003; Killcross and Coutureau, 2003; Valentin et al., 2007; Yin et al., 2004, 2005). Although our ventral striatal findings are consistent with the possibility of interactions between Pavlovian and instrumental control systems, the absence of any correlations between performance decrements and activity in brain systems known to be involved in goal-directed or habitual control do not lend support for the explicit monitoring theory (at least in relation to the present study).

It is also important to note that although our present findings support the role of aversion-related mechanisms in performance decrements, we cannot rule out possible contributions of additional maliferous mechanisms in mediating performance decrements under other task conditions or contexts. It remains an open question whether similar mechanisms play a role in driving performance decrements in the presence of stressors other than large incentives, such as audience effects or competition. It is entirely possible that no single mechanism will account for all instances of the choking effect.

Our findings in the striatum also have implications for economic theories of choice. Koszegi and Rabin (2006) have suggested that we do not define our reference point for the value of decisions and actions in the absolute terms specified by the environment; instead we set an internal reference point based on our expectations of a task outcome. The rapid switching of ventral striatum, and loss sensitivity at the time of motor action that we have shown here, suggests that the ventral striatum might play a role in encoding such an endogenous reference point. In a sense, when participants see they are playing for $100, they view this money as being endowed to them and theirs to lose. When they actually perform the task, their loss aversion is revealed and manifested as decrements in performance.

Two recent behavioral economics studies postulate that reference dependent utility could influence performance decrements in the context of professional athletics. In one of these studies the authors examined millions of putts from professional golfers and suggested that the par score of a hole served as a reference point for players; with putts being less accurate when attempting shots below par (Pope and Schweitzer, 2011). Another study examined penalty kick shootouts of soccer games (Apesteguia and Palacios, 2010). This study proposed that the score of the shootout served as a player's reference point and leading or lagging in score had an influence on performance; with those lagging in score performing worse than those leading. These studies provide interesting insights into the possibility of an endogenous reference point of value influencing skilled task performance. However, these hypotheses are difficult to generalize because the contexts in which the sports are played are highly variable and the data lack a degree of experimental control. Furthermore, it was impossible to directly isolate players' endogenous reference point of value because psychological and physiological measures were not available in these data sets. Instead, these studies only infer possible mechanisms used to define reference points during task performance.

Our study provides direct behavioral and neural evidence of the mechanism responsible for encoding an endogenous reference point during skilled task performance for incentives. It is important to realize that the hypothesis of a reference dependent encoding of value, and exactly how this reference point is defined, was informed and driven by our initial imaging analysis (experiment 1). Without this fMRI analysis, one would simply expect, as we did initially, that increasing incentives for task performance are encoded solely as increasing potential gains. In contrast, our fMRI analysis informed the hypothesis that the brain encodes increasing potential gains when the amount of incentive is initially presented, however when actually performing the task potential gains are reframed in terms of losses. This neurally informed hypothesis was confirmed using a separate experiment (experiment 2) in which we related a behavioral measure of loss aversion to task performance. In this way we were able to uncover the specific mechanism involved in encoding an endogenous reference point of value, and shed light on how it influences skilled task performance. This study highlights how neuroscience methods can provide insights of economic behavior: one of the major goals of the burgeoning field of neuroeconomics.

Economists have long pondered the question of how best to design incentive contracts that pay workers just enough to fully maximize their performance (Smith, 1776). Standard models of these contracts assume that both the principal (manager) and agent (worker) act rationally and in a fashion that maximizes their individual utility (Laffont and Martimort, 2001). Under this assumption it follows that a worker's performance should monotonically increase with pay. The results of our study illustrate that performance responses to incentives are far more nuanced, and beyond the fine balance between performance and pay are loss aversion and detrimental performance effects.

Our findings also have implications for understanding the nature of performance decrements in situations where skilled motor acts need to be performed under conditions of high stakes, such as in sporting competitions (Jordet and Hartmen, 2008; Smith et al., 2003), or even in life and death situations such as surgery or the operation of machinery in hazardous environments. We have shown that people with less striatal sensitivity to incentive (i.e., the most stable neural response over the range of incentives) perform high stakes tasks with more proficiency. With this in mind, it is plausible that the implementation of explicit cognitive strategies designed to focus an individual away from the prospect of failure could serve to stabilize neural activity and mitigate potential performance decrements.

## Experimental Procedures

### Experimental Setup

Stimulus presentation and behavioral data acquisition were achieved using custom designed MATLAB (http://www.mathworks.com) and C++ programs implementing the OpenGL (Silicon Graphics) graphics libraries. During functional magnetic resonance (fMRI), visual feedback of targets and hand position were presented via a projector positioned at the back of the room. Participants viewed a reflection of the projector image (800 × 600 pixels) in a mirror attached to the scanner head coil. This system allowed us to generate virtual images and manipulate visual feedback.

Direct view of the arm was obscured because participants were positioned in the scanner head-first-supine, and the display mirror blocked their view. A Vicon motion tracking system (MX Ultranet system, with 4 MX40+ cameras; Oxford Metrics, Oxford, UK) was used to record the motion of an infrared reflective maker attached to the right index finger. During experiments, these signals were sent to our custom designed software for visual real-time feedback of participants' hand position. The position signals were also recorded for further offline analysis. Participants' arm movements were confined to the coronal plane, and visual feedback of these movements was presented in 2D on the visual display.

### Experimental Procedures

#### Participants

All participants were right handed, and were prescreened to exclude those with a prior history of neurological or psychiatric illness. The California Institute of Technology Institutional Review Board approved this study, and all participants gave informed consent.

Eighteen participants (mean age, 26; age range, 19–35; seven females) took part in the first experiment (experiment 1). Of these 18 participants 12 returned for a subsequent test of behavioral loss aversion.

For the follow-up experiment (experiment 2), an additional 20 participants were recruited (mean age, 23; age range, 19–30; nine females), however, they did not perform the experiment in the fMRI scanner. They performed the motor task in a mock scanning environment to duplicate the postural constraints of the actual scanner. They also performed loss aversion and risk aversion tasks.

#### Motor Task

The experiment was comprised of three phases that took place on two consecutive days. On the first day, participants practiced control of the spring-mass system (training phase). For a more detailed description of the spring-mass system see the Supplemental Information. After the training phase, we determined participants' rates of success at various target sizes (thresholding phase). On the second day, participants controlled the spring-mass system with the purpose of obtaining reward (testing phase). Both the training and thresholding phases took place in a mock scanner to replicate the posture necessary in the scanning environment. The testing phase took place in the fMRI scanner. Prior to the experiment, participants were told they would receive a show-up fee of $40 dollars, and that at the end of the experiment one trial would be randomly selected from the testing phase and a payment made according to their actual performance on that trial. This is a standard procedure used in behavioral economics, which ensures that participants evaluate each trial independently.

The training phase was comprised of 500 trials. A trial began when a participant put her hand cursor over the start position and ended after 2 s. At the end of the trial, the cursors flashed green if the scoring criteria were met and red otherwise. The target size was 50^2^ mm throughout the training phase. The thresholding phase was the same as the training in all respects, except that it was comprised of 200 trials of varying size. Target sizes ranged from 10^2^ mm to 55^2^ mm in increments of 5^2^ mm. Each target size was randomly presented 20 times. From this data we obtained a psychometric curve that represented participants' performance over a range of target sizes.

Finally, during the testing phase participants were scanned with fMRI while controlling the spring-mass system for reward. Participants performed trials for a range of incentives (i.e., $0, $5, $25, $50, $75, $100) and at two difficulty levels (i.e., easy, hard). The difficulty levels were tailored to each participant using their respective psychometric curves. The easy level corresponded to the target size at which participants have an 80% success rate, and hard coincided with a 60% success rate. Each treatment was randomly presented 25 times for a total of 300 trials. At the beginning of each trial, participants were shown a message indicating the amount of incentive they were playing for (e.g., Win $50) (jittered duration 2–5 s). They then performed the motor task, with the same success criteria as before (duration 2 s), and were shown the trial outcome (1 s). At the end of the experiment a single trial was selected at random and the participant was paid based on performance on that trial.

#### Loss Aversion Task

This task was performed outside the fMRI scanner. Participants received an initial endowment of $40 in cash (this amount was separate from their show-up fee) and were told that at the end of the experiment one trial would be randomly selected and a payment made according to their actual decision during the experiment. Participants were told that their $40 endowment was given to them so that they could pay any eventual losses at the end of the experiment. Any net amount from the endowment that remained after subtracting a loss was theirs to keep, and similarly any eventual gain earned in the experiment was added on top of the initial endowment.

The experiment consisted of 512 trials. During the task participants were asked to accept or reject a series of mixed gambles with equal (50%) probability of winning or losing a variable amount of money. These gambles were presented on a computer screen as the prospective outcomes of a coin flip, and participants indicated their willingness to take the gamble by key press. Trials were self-paced. Each trial was uniquely and randomly sampled from a gains/losses matrix with potential gains ranging from +$10 to +$40 and potential losses from −$5 to −$20 in increments of $2. This task is the same as that used by Tom et al. (2007).

#### Risk Aversion Task

Participants were also tested on their general risk attitude (independent from loss aversion) using a series of monetary gambles that included only gains. In each trial, each participant was presented with the choice either to accept a safe option (i.e., a variable sure monetary amount) or to play a risky gamble (i.e., flip a coin to receive a larger amount of money or get nothing). The sure amount was either $10, $15, or $20. Corresponding gambles ranged from $16 to $27, $26 to $37, and $36 to $47 respectively (in increments of $1). Each trial was presented six times (216 trials in total) in random order. At the end of the experiment a trial was randomly selected and a payment was made according to the participants' decision and a random outcome. This is an adaption of the risk task developed by Holt and Laury (2002).

#### MRI Protocol

A 3 Tesla Siemens Trio (Erlangen, Germany) scanner and standard radio frequency coil was used for all the MR scanning sessions. To reduce the possibility of head movement related artifact, participants' heads were securely positioned with foam position pillows. High resolution structural images were collected using a standard MPRAGE pulse sequence, providing full brain coverage at a resolution of 1 mm × 1 mm × 1 mm. Functional images were collected at an angle of 30° from the anterior commissure-posterior commissure (AC-PC) axis, which reduced signal dropout in the orbitofrontal cortex (Deichmann et al., 2003). Forty-five slices were acquired at a resolution of 3 mm × 3 mm × 3 mm, providing whole-brain coverage. A one-shot echo-planar imaging (EPI) pulse sequence was used (TR = 2800 ms, TE = 30 ms, FOV = 100 mm, flip angle = 80°).

### Data Analysis

#### Behavioral Performance Analysis

To account for differences in behavioral performance variance between participants (which contributed to extraneous variance in the aggregate data) we Z-scored participants' performance measurements. To do this, each participants' measures of performance were separately standardized (Z-scored) across incentive categories. Z-scoring was achieved by taking a performance level in an incentive category and subtracting it from the mean performance for all incentive categories divided by the standard deviation. This preserved the relative ordering of performance levels across incentives. Z-scoring is a widely used method for normalizing ratings data between subjects that provides a standard performance scale over which to evaluate group behavioral data (Martin and Bateson, 1993).

Due to differences in participants' subjective value for monetary incentives, participants exhibited peak performance over the range of incentive levels (Figure 3B) (Ariely et al., 2009), therefore averaging performance at the presented incentive bins would attenuate the effect of peaked responses to incentives. To illustrate that group performance peaked and then dropped with increasing incentives, we classified the presented incentives as either being at the extremes of incentives or in the middle range of incentives. Rewards in the middle range of incentives were classified as those between 5% and 95% of the range of incentives (middle range of incentives: [$25, $50, $75]), while rewards at the extremes of incentive were those outside this range (low extreme: [$0, $5]; high extreme: [$100]).

To ensure that the results we obtained from our *Z*-scored performance data were not an artifact of our normalization approach, we simulated 10,000 experiments each comprised of 18 subjects (the number of subjects in our fMRI data set) wherein performance levels were sampled from a normal distribution (mean = 70%, std = 10%). When performing a t test comparing the Z-scored performance at the extremes of incentive ($0, $5, $100) with the middle range of incentive ($25, $50, $75) we found that significance was reached at the 5% level in less than 3% of simulations as would be expected for an unbiased sample at the 5% significance level. Furthermore, in a subsequent analysis we found that 0 out of 10,000 of these simulations resulted in a significant ANOVA at p < 0.05 and significant increases and decreases in Z-scored performance across three incentive categories (low: $0,$5; medium: $25, $50, $75; high: $100).

#### Image Processing and fMRI Statistical Analysis

The SPM5 software package was used to analyze the fMRI data (Wellcome Department of Imaging Neuroscience, Institute of Neurology, London, UK). A slice-timing correction was applied to the functional images to adjust for the fact that different slices within each image were acquired at slightly different points in time. Images were corrected for participant motion, spatially transformed to match a standard echo-planar imaging template brain, and smoothed using a 3D Gaussian kernel (6 mm FWHM) to account for anatomical differences between participants. This set of data was then analyzed statistically.

The general linear model (GLM) was used to generate voxelwise statistical parametric maps (SPMs) from the fMRI data. To generate the results presented in the main text we created a GLM that included categorical events at the time of incentive presentation and separate events for each combination of motor task conditions (incentive level, difficulty, performance). The incentive presentation event was modeled with a duration lasting the length of incentive presentation (2–5 s), whereas the motor task event was modeled with a fixed duration of 2 s. Because there were six incentive levels ($0, $5, $25, $50, $75, $100), two difficulty levels (easy, hard), and two performance outcomes (successful, unsuccessful), this resulted in 24 categorical events to model all condition combinations of the motor task. Including the incentive presentation event, a grand total of 25 categorical events were modeled. We also included incentive level as a parametric modulator at the time of the incentive presentation event. In addition, regressors modeling the head motion as derived from the affine part of the realignment produce were included in the model.

With this model we tested brain areas in which activity was correlated with incentive level at the time of incentive presentation. This was done by creating contrasts with the aforementioned parametric modulator for incentive at the time of incentive presentation. We also examined areas in which activity was correlated with incentive level at the time of the motor task. This was done by creating linear contrasts for the motor task conditions at the varying incentive levels (separated among difficulty levels and performance outcomes). To increase statistical power these contrasts (Figure 4) were computed for trials collapsed across difficulty levels; and to control for actual performance they were computed for only those trials in which participants were successful.

We created a separate GLM to test for differences in brain activity between performance outcomes (i.e., unsuccessful and successful trials) during the motor task, and activity showing an interaction between incentives and performance during the motor task. This model included a categorical event at the time of incentive presentation and separate events at the time of the motor task for unsuccessful and successful trials. Each of these categorical regressors included a parametric modulator corresponding to the level of incentive presented. The main effect regressors for unsuccessful and successful trials were subtracted to create contrasts showing the differences between successful and unsuccessful trials. To create the interaction contrast (Figure 7) we subtracted the incentive parametric modulators, at the time of the motor task, for unsuccessful and successful trials.

#### Analysis of Behavioral Loss Aversion Data

To estimate participants' loss aversion we used a parametric analysis. We expressed participants' utility function *u* for monetary values *x* as

$$u\left(x\right)=\{\begin{array}{cc} x & x\geq 0\\ \text{λ}x & x<0 \end{array}.$$

This formulation is similar to that introduced by Tversky and Kahneman (1992), except we assumed that *u*(*x*) was piece-wise linear over the range of potential gains and losses presented to participants (an assumption that is commonly employed [Frydman et al., 2011; Gachter et al., 2007; Tom et al., 2007]). In this formulation, λ represents the relative weighting of losses to gains, and λ > 1 indicates that losses loom larger than equal-sized gains.

Assuming participants combine probabilities and utilities linearly the expected utility of a mixed gamble can be written as *U*(*G*, *L*) = 0.5 *G* + 0.5 λ*L*, where *G* and *L* are the respective gain and loss of a presented risky option. The probability that a participant chooses to make a gamble is given by the softmax function

$$P\left(G,L\right)=\frac{1}{1+\exp\left(-\tau U\left(G,L\right)\right)},$$

where τ is a temperature parameter representing the stochasticity of a participant's choice (τ = 0 means choices are random).

We used maximum likelihood to estimate parameters λ and τ for each participant, using 512 trials of mixed gambles (*G*,*L*) with participant response *y* ∈ {0,1}. Here *y = 1* indicates that the participant chose to make a gamble. This estimation was performed by maximizing the likelihood function

$$\sum_{k=1}^{512}y_{i}\;\log\left(P\left(G,L\right)\right)+\left(1-y_{i}\right)\;\log\left(1-P\left(G,L\right)\right)$$

using Nelder-Meas Simplex Method in Matlab 2008b.

Median parameter estimates for experiment 1 (n = 12) were λ = 2.09 (IQR 1.09) and τ = 0.70 (IQR 0.27). Median parameter estimates for experiment 2 (n = 20) were λ = 2.20 (IQR 0.75) and τ = 0.60 (IQR 0.44).

#### Analysis of Behavioral Risk Aversion Data

Because participants' risk aversion was tested using a separate set of behavioral choices we used a separate parametric analysis for estimation. The risk aversion task only included potential gains *x*, and we expressed participants' utility *u* as

$$u\left(x\right)=x^{\text{α}}\;x\geq 0.$$

This formulation is from prospect theory and is commonly used to characterize utility in the gain domain (Tverskey and Kahneman, 1992). It captures participants decreasing sensitivity to potential gains as the magnitude of gains increases. The parameter α represents the degree of a participants' risk aversion (α = 1 characterizes risk neutrality; α < 1 risk aversion; α > 1 risk seeking behavior).

A participants' difference in expected utility for mixed gambles comprised of a risky option (*G*,0) and a sure option *S* is expressed as *U*(*G*, *S*) = 0.5 *G*^α^ − *S*^α^. The probability that a participant chose to make a gamble is

$$P\left(G,S\right)=\frac{1}{1+\exp(-\text{τ}U\left(G,S\right)}.$$

As in the case of the loss aversion data, we used numerical optimization to estimate the parameters α and τ for each participant by maximizing the likelihood function

$$\sum_{i=1}^{216}y_{i}\;\log\left(P\left(G,S\right)\right)+\left(1-y_{i}\right)\;\log\left(1-P\left(G,S\right)\right).$$

Median parameter estimates for experiment 2 (n = 20) were α = 0.83 (IQR 0.20) and τ = 2.46 (IQR 1.70). Risk aversion was not estimated for participants in experiment 1 because they did not perform the risk aversion task.

## Figures and Tables

**Table 1:** Correlations between Prospect Theory Parameters and Performance for Participants in the Follow-Up Experiment

	Correlation with Loss Aversion (λ); n = 20		Correlation with Risk Aversion (α); n = 20	
	Performance at $100	Incentive Resulting in Peak Performance	Performance at $100	Incentive Resulting in Peak Performance
Easy difficulty	r = 0.31	r = 0.26	r = 0.17	r = 0.22
	p = 0.18	p = 0.27	p = 0.47	p = 0.35
Hard difficulty	r = 0.61	r = 0.44	r = 0.18	r = 0.10
	p = 0.004	p = 0.05	p = 0.45	p = 0.67

Each element of this table indicates the significance of a correlation between participants' loss aversion and risk aversion and performance measures at each difficulty level.

## Supplemental Information

Document S1. Figures S1–S5, Tables S1–S4, and Supplemental Experimental Procedures
