# Supplementary material for: Neural Mechanisms Underlying Paradoxical Performance for Monetary Incentives Are Driven by Loss Aversion
Source: Neuron. 2012 May 10;74(3):582–94. doi: 10.1016/j.neuron.2012.02.038 (PMC3437564; doi:10.1016/j.neuron.2012.02.038)
Supplement: Document S1. Figures S1–S5, Tables S1–S4, and Supplemental Experimental Procedures [file mmc1.pdf]

Neuron, Volume *73*

Supplemental Information

Neural Mechanisms Underlying Paradoxical Performance for Monetary Incentives Are Driven by Loss Aversion

Vikram S. Chib, Benedetto De Martino, Shinsuke Shimojo, and John P. O'Doherty

INVENTORY OF SUPPLEMENTAL INFORMATION

Supplemental Data

Figure 3: Behavioral performance during scanning; associated with

- **Figure S2**: Kinematic trajectory metrics

Figure 4: fMRI results; associated with

- **Figure S1**: Simulations of fMRI design and additional whole brain analyses
- **Table S1**: Regions with a significant increase in fMRI signal for increasing incentives at the time of incentive presentation
- **Table S2**: Regions with a significant decrease in fMRI signal for increasing incentives at the time of the motor task
- **Table S3**: Locations of significant activation in a conjunction analysis between areas showing a decrease in signal for increasing incentives at the time of motor performance and an increase in signal for increasing incentives at the time of incentive presentation

Figure 5: Loss aversion across participants; associated with

- **Figure S3**: Independent replication of neural sensitivity / loss aversion correlation

Figure 6: A prediction error model does not describe neural responses to incentives; associated with

- **Figure S4**: fMRI results for easy and hard conditions during the motor task.

Figure 7: Interaction between task performance (unsuccessful/successful trials) and incentive level ($0, 5, 25, 50, 75, 100); associated with

- **Figure S5**: Conjunction between areas showing a decrease in signal for increasing incentives at the time of motor performance, an increase in signal for increasing incentives at the time of incentive presentation, and an interaction between task performance and incentive level.
- **Table S4**: Regions with a significant interaction between task performance (success/failure) and increasing incentives at the time of the motor task

Supplemental Experimental Procedures

Motor Task Details

Simulation of the Spillover of the Neural Response from Incentive Presentation to the Motor Task; associated with **Figure S1A,B**

Analysis of the Physiological Spillover Between Incentive Presentation and the Motor task; associated with **Figure S1C**

Whole Brain Functional Imaging Analysis; associated with **Figure S1E**

fMRI Region of Interest Analysis and Neural Sensitivity to Incentive; associated with **Figure S1D**

Kinematic Trajectory Analysis; associated with **Figure S2**

Independent Replication of Neural Sensitivity / Loss Aversion Correlation; ; associated with **Figure S3**

Supplemental References

Supplemental Data

Figure S1: Simulations of fMRI design and additional whole brain analyses

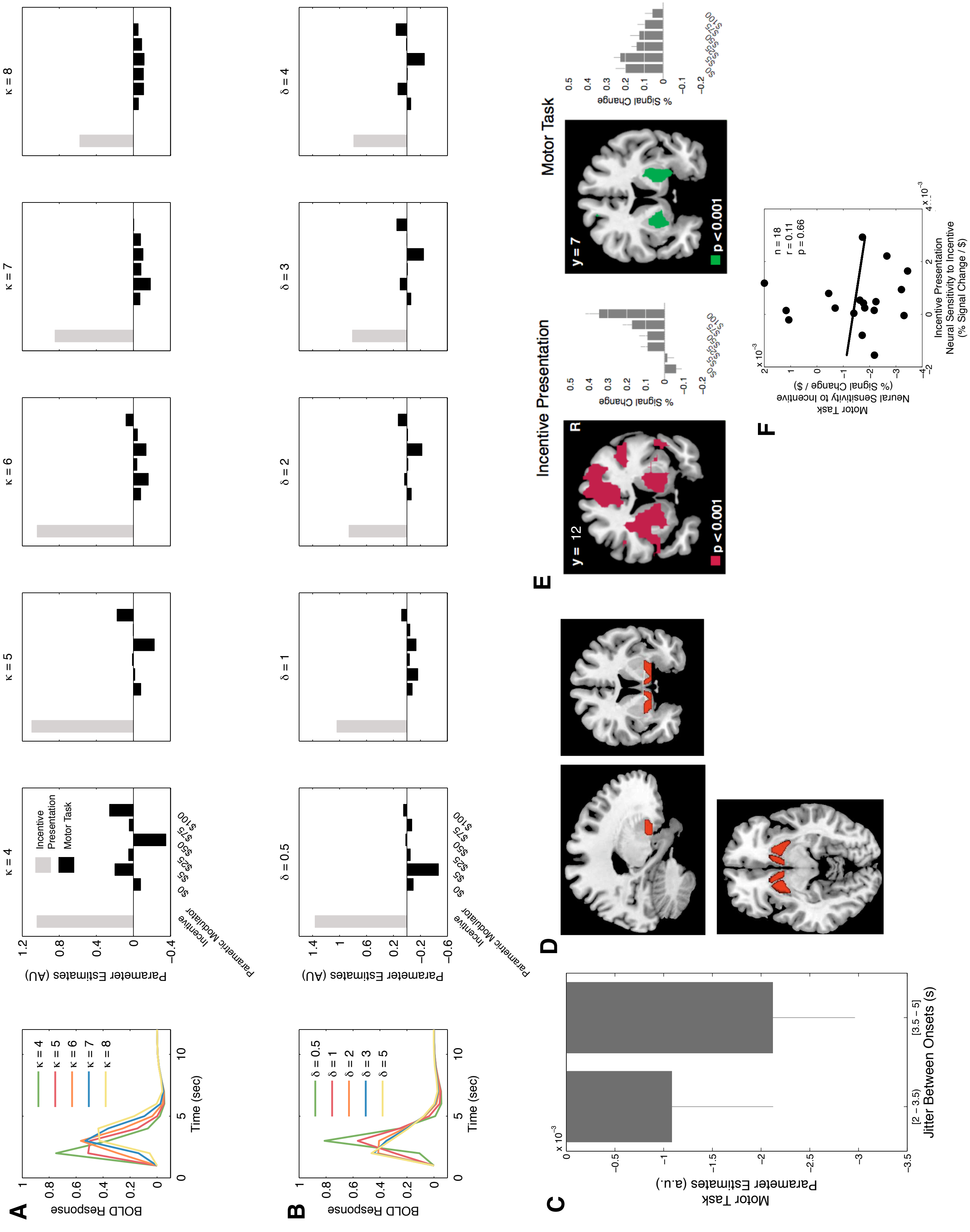

Figure S1.
Simulation of the effects of different BOLD responses on the separability of responses to incentive presentation and the motor task (see Supplemental Experimental Procedures for details of these simulations). In particular, we tested if the deactivations we observed experimentally during the motor task were the result of a spillover of hemodynamic responses from the incentive presentation phase. For these simulations we examined how differing simulated hemodynamic response functions (HRF) of **A**) variable delays to peak (κ = 4, 5, 6, 7, 8 seconds) and **B**) dispersions (δ = 0.5, 1, 2, 3, 4 seconds), for the incentive presentation phase, influenced measured responses during the motor task phase (using the same GLM used to analyze the actual BOLD experimental data). The bar graphs show that the incentive presentation modulators (grey bars) were accurately modeled with our GLM, and that there was minimal spillover of response in the motor task conditions (black bars), as indicated by parameter estimates being randomly spread around 0. Furthermore, the parameter estimates for the motor task conditions did not follow a general trend of increasing deactivation with magnitude of incentive. These results confirm that the striatal deactivation observed during the motor task (Figure 3a of the main text) was not the result of a spillover of hemodynamic responses from the incentive presentation phase.

C) To test that the deactivations we observed during the motor task were not the result of a physiological spillover from the incentive presentation, we performed another analysis in which we examined neural responses in trials binned by their jitter between incentive presentation and task performance (i.e., small jitter, [2 s- 3.5 s); large jitter, [3.5 s - 5 s]). The rational being that if deactivations during the motor task were the result of physiological crosstalk from the incentive presentation event, longer jitter duration trials would have less propensity to corrupt neural responses during the motor task, and would result in a trend of diminished effect sizes for trials with longer jitter durations. Consistent with the imaging results presented for all trials, neural responses decreased with increasing incentives at the time of the motor task (i.e., negative parameter estimates). Most importantly we did not find a significant difference between parameter estimates for short and long jitter duration trials ($p < 0.42$). These results suggest that the neural deactivations observed during the motor task are not the result of physiological crosstalk from the incentive presentation event. For further details of this analysis see the Supplemental Experimental Procedures.

D) Illustration of region of interest (ROI). All results reported in the main text are with a corrected significance threshold of $P<0.05$, based on small-volume correction within an anatomically defined ROI bilaterally encompassing the ventral striatum (incorporating the nucleus accumbens extending into the ventral parts of the putamen).

E) Additional fMRI results (See Supplemental Experimental Procedures for details). A whole brain search for regions underlying performance responses to incentives found results in correspondence with the those reported in the main text. Activity in ventral striatum was positively correlated with incentive level at the time of incentive presentation [x = 12; y = 12; z = -6], and negatively correlated with incentive level at the time of the motor task [x = 21; y = 7; z = -9]. All contrasts are significant at $p<0.05$, small volume corrected. Percent signal changes were extracted from average voxel activity in a sphere 20 mm in radius, centered at [x = -0.4, y = 6.1, z = 1.5]. These coordinates were chosen because they were previously found to commonly encode potential gains and losses [1].

F) Neural sensitivities to incentive during incentive presentation and execution of the motor task we not significantly correlated.

Significant Regions in Main Imaging Analyses

For the contrasts reported in the main text, we report results in a priori regions of interest previously identified in neuroimaging studies on decision-making and reward (striatum, dorsolateral prefrontal cortex, orbitomedial prefrontal cortex, anterior cingulate cortex) and goal directed attention and arousal (ventrolateral prefrontal cortex, insula, premotor cortex, supplementary motor area, parietal cortex, cingulate, amygdala) [1-6].

Table S1. Regions with a significant increase in fMRI signal for increasing incentives at the time of incentive presentation (*p*<0.001 uncorrected). At the time of incentive presentation, no regions showed decreasing activity for increasing incentives. Statistically significant activations are those found in apriori regions of interest FWE p<0.05, and those regions that survive whole-brain correction for multiple comparisons at P < 0.05 (indicated with *).

**Region**	**Laterality**	**x**	**y**	**z**	**T value**	**Correlation between Neural Sensitivity and Behavioral Performance at $100**
Striatum (nucleus accumbens, ventral putamen) *	R	12	12	-6	6.51	r = 0.22 ; p = 0.38
Striatum (nucleus accumbens, ventral putamen) *	L	-21	15	-3	5.59	
Dorsolateral Prefrontal Cortex *	R	42	42	33	6.95	r = 0.10 ; p = 0.69
Insula *	R	30	24	-6	6.00	r = 0.41; p = 0.09
Insula *	L	-39	15	3	6.20	r = 0.43; p = 0.07
Anterior Cingulate	M	6	3	33	5.34	r = 0.33 ; p = 0.18
Supplementary Motor Area *	R	9	15	48	7.20	r = 0.29 ; p = 0.23
Premotor Area *	R	30	6	51	8.07	r = 0.02; p = 0.93

Region	Laterality	x	y	z	T value	Correlation between Neural Sensitivity and Behavioral Performance at $100
Premotor Area *	L	-39	-6	48	5.32	r = 0.40 ; p = 0.10
Parietal Cortex (BA 7) *	R	39	-48	54	6.98	r = 0.24 ; p = 0.33
Parietal Cortex (BA 7) *	L	-24	-57	57	7.05	r = 0.40 ; p = 0.10
Occipital Cortex (BA 18)	R	21	-93	9	6.63	r = 0.30 ; p = 0.23
Cerebellum (Posterior Lobe)	L	-30	-63	-15	11.08	r = 0.02 ; p = 0.93

Table S2. Locations of significant activation in a conjunction analysis between areas showing a decrease in signal for increasing incentives at the time of motor performance and an increase in signal for increasing incentives at the time of incentive presentation ($p<0.001$ uncorrected). Statistically significant activations are those found in apriori regions of interest FWE $p<0.05$, and those regions that survive whole-brain correction for multiple comparisons at $P < 0.05$ (indicated with *).

Region	Laterality	x	y	z	T value
Striatum (nucleus accumbens, ventral putamen) *	R	21	9	-9	4.01*
Striatum (nucleus accumbens, ventral putamen) *	L	-18	6	-6	3.84*
Thalamus	R	9	-19	0	4.99

Table S3. Regions with a significant decrease in fMRI signal for increasing incentives at the time of the motor task (p<0.001 uncorrected). At the time of the motor task, no regions showed increasing activity for increasing incentives. Statistically significant activations are those found in apriori regions of interest FWE $p<0.05$, and those regions that survive whole-brain correction for multiple comparisons at $P < 0.05$ (indicated with *).

<table>
<tr><th>Region</th><th>Laterality</th><th>x</th><th>y</th><th>z</th><th>T value</th><th>Correlation between Neural Sensitivity and Behavioral Performance at $100</th></tr>
<tr><td>Striatum (nucleus accumbens, ventral putamen) *</td><td>R</td><td>21</td><td>9</td><td>-9</td><td>4.15*</td><td rowspan="2">r = 0.70 ; p = 0.001</td></tr>
<tr><td>Striatum (nucleus accumbens, ventral putamen) *</td><td>L</td><td>-18</td><td>6</td><td>-6</td><td>3.89*</td></tr>
<tr><td>Posterior Parietal Cortex (BA 39)*</td><td>R</td><td>48</td><td>-72</td><td>33</td><td>5.01</td><td>r = 0.23 ; p = 0.34</td></tr>
<tr><td>Thalamus</td><td>R</td><td>9</td><td>-19</td><td>0</td><td>4.99</td><td></td></tr>
</table>

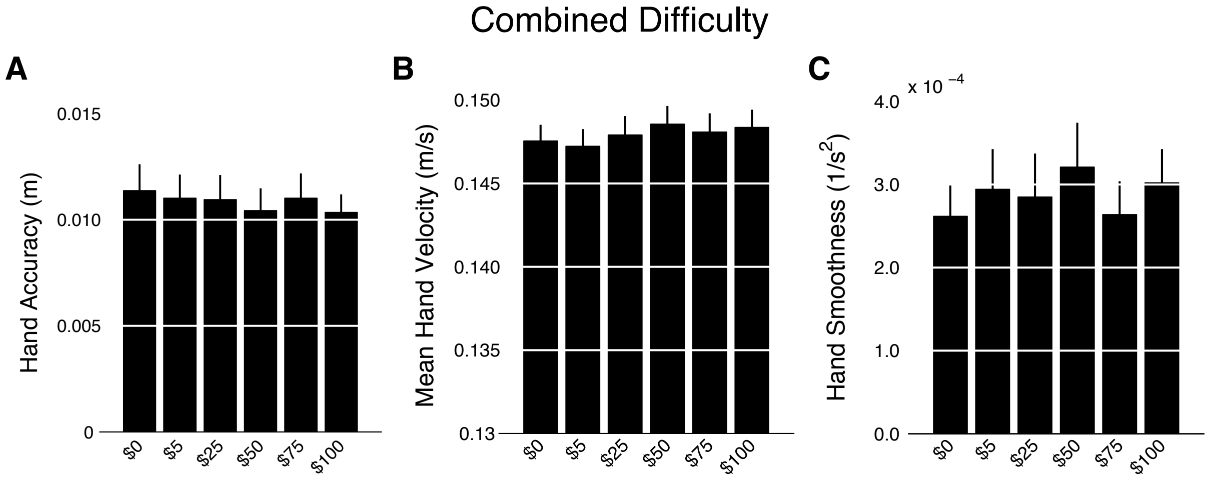

Figure S2. Kinematic trajectory metrics. Plots shown are for trials in which participants were successful, collapsed across difficulty levels. No significant differences were found between these measures across incentives levels, indicating participants had consistent kinematic motor behaviors regardless of incentive level. **A**) Hand accuracy (F(5, 85) = 1.75, p = 0.13). **B**) Mean hand velocity (F(5, 85) = 1.58, p = 0.18). **C**) Hand smoothness (F(5, 85) = 0.31, p = 0.90). For details of these metrics see the Supplemental Experimental Procedures.

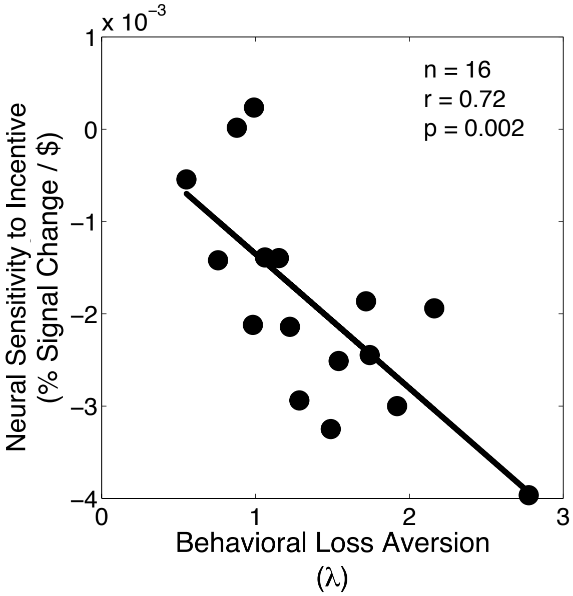

Figure S3. Replication of neural sensitivity/behavioral loss aversion correlation. Individual participants' behavioral loss aversion was significantly correlated to their striatal sensitivity to incentive. For details of this experiment see Supplemental Experimental Procedures.

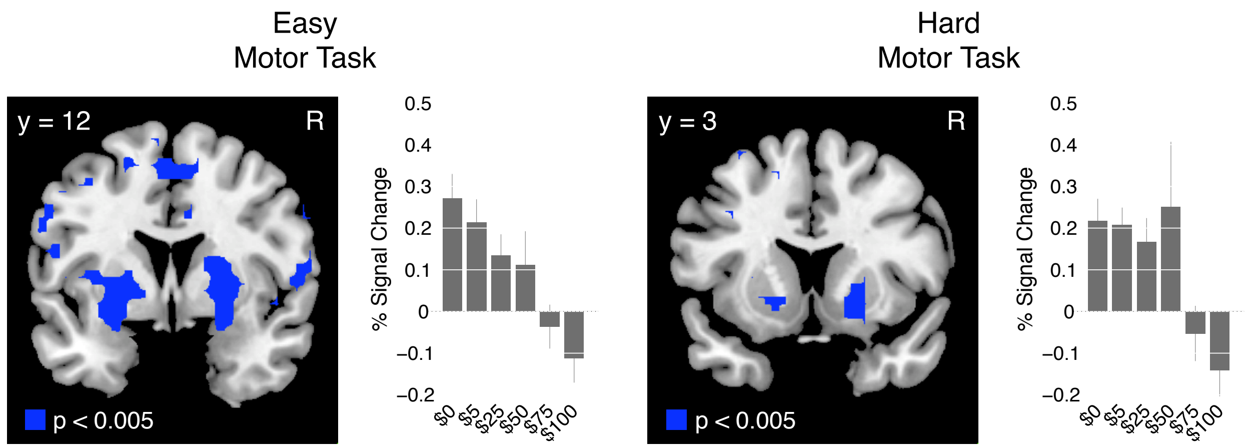

Figure S4. fMRI results for easy and hard conditions during the motor task. Activity in ventral striatum was negatively correlated with incentive level at the time of the motor task (easy: [x = 21; y = 12; z = -6]; hard: [x = 24; y = 3; z = -9]). All contrasts are significant at $p<0.05$, small volume corrected. As in the contrasts in the main text, all percent signal change data was extracted from average voxel activity in a sphere 20 mm in radius, centered at [x = -0.4, y = 6.1, z = 1.5].

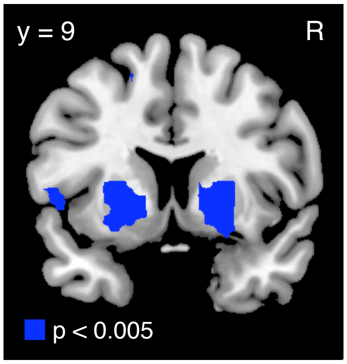

Figure S5. Conjunction between areas showing a decrease in signal for increasing incentives at the time of motor performance, an increase in signal for increasing incentives at the time of incentive presentation, and an interaction between task performance and incentive level. This analysis found a common region in ventral striatum [x = 21; y = 9; z = -12].

Table S4. Regions with a significant interaction between task performance (success/failure) and increasing incentives at the time of the motor task (p<0.001 uncorrected). The only area that was significant after small volume correction was bilateral ventral striatum.

We defined statistically significant activations as those found in apriori regions of interest FWE p<0.05, and those regions that survive whole-brain correction for multiple comparisons at P < 0.05 (indicated with *).

Region	Laterality	x	y	z	T value
Striatum (nucleus accumbens, ventral putamen) *	R	27	0	0	6.96
Striatum (nucleus accumbens, ventral putamen) *	L	-27	3	-3	5.05
Dorsal premotor cortex	L	-39	12	45	4.70
Thalamus	R	12	-21	0	5.62

Supplemental Experimental Procedures

Motor Task Details

During the experiment participants performed a highly skilled motor task: control of a virtual spring mass system [7]. This task was chosen because the spring-mass has a well-defined set of equations, and its control serves as a benchmark of performance in modern control theory [8]. Moreover, this dynamic system was completely novel to participants, which allowed us to evaluate performance uncorrupted by previous experience or expertise.

The spring mass system had the equations of motion:

$$M_o \ddot{r}_o + K_o(r_o - r_h) = 0$$

where M_o was the object mass and K_o was the object spring stiffness, and $r_o = [x_o, y_o]'$ and $r_h = [x_h, y_h]'$ are the positions of the object and the hand respectively. The state space representation of this equation was obtained by defining the object state variables $q_1 = x_o$, $q_2 = y_o$, $q_3 = x_o'$, $q_4 = y_o'$ and $\varepsilon_{11} = \varepsilon_{12} = K_o/M_o$, $\varepsilon_{21} = \varepsilon_{22} = K_o/M_o$.

$$\begin{bmatrix} \dot{q_1} \\ \dot{q_2} \\ \dot{q_3} \\ \dot{q_4} \end{bmatrix} = \begin{bmatrix} 0 & 0 & 1 & 0 \\ 0 & 0 & 0 & 1 \\ -\varepsilon_{11} & -\varepsilon_{12} & 0 & 0 \\ -\varepsilon_{21} & -\varepsilon_{22} & 0 & 0 \end{bmatrix} \begin{bmatrix} q_1 \\ q_2 \\ q_3 \\ q_4 \end{bmatrix} + \begin{bmatrix} 0 & 0 \\ 0 & 0 \\ \varepsilon_{11} & \varepsilon_{12} \\ \varepsilon_{21} & \varepsilon_{22} \end{bmatrix} \begin{bmatrix} x_h \\ y_h \end{bmatrix}$$

The control input of the system was $[x_h, y_h]$ the position of the hand. These equations assumed a zero rest length of the spring. We defined M_o= 3kg and K_o =120 Nm^{-1} as in Dingwell and Mussa-Ivalid [7]. The state equations for the system were integrated in real-time to compute the instantaneous position of the object for each corresponding position of the hand. Reaching movements were always initiated with the hand and object both at rest at the same position. During trials participants were instructed to move both their hand and the mass from a start position to a target 20 cm away. A successful trial consisted of both the hand and mass cursors being placed in the target, below a velocity of 0.02 m/s, after two seconds.

Simulation of the Spillover of Neural Response from Incentive Presentation to the Motor Task

To ensure that the deactivations we observed during the motor task were not the result of spillover from the BOLD response of the incentive presentation phase, we performed a simulation of this effect. This simulation examined whether increasing hemodynamic responses elicited at the time of incentive presentation could, by virtue of a spillover to the motor phase, account for the neural deactivation effects we observed in the ventral striatum during the motor task.

We first simulated BOLD time courses using a trial vector of onsets from a representative participant. BOLD time courses were generated by convolving a trial vector of impulse responses taking place at the onset of incentive presentation with a family of HRFs. To incorporate differences in neural response for differing levels of incentive, impulse responses were scaled with respect to the group normalized percent signal change shown in Figure 3A of the main text. In order to establish the robustness of our simulation to variations in the time to peak and the shape of the hemodynamic response functions we used a family of hypothetical HRF responses that had varying delays (κ = 4, 5, 6, 7, 8 seconds) and dispersions (δ = 0.5, 1, 2, 3, 4 seconds). This range of HRF parameters is similar to those previously reported as being within the range of physiologically plausibility [9].

To model the simulated neural data we created a GLM for the same representative participant using condition onsets for the incentive presentation and motor task phases. The following conditions and onsets were included in the GLM: incentive presentation; easy and hard motor task conditions that were successful; and easy and hard motor task conditions that were missed. The motor task conditions were also divided by their incentive levels (i.e., $0, 5, 25, 50, 75, 100). In this way 25 conditions were modeled. We also included incentive level as a parametric modulator at the time of incentive presentation. This GLM was identical to that used to model actual experimental data, except it did not include motion regressors.

It is important to note that our simulated time courses did not model neural responses during the motor task. However, we did model these onsets in our GLM. This allowed us to examine the extent to which the neural responses of the incentive presentation events could have spilled into the motor task conditions. In particular, we tested our simulated data for similar effects to those seen in our actual data, at the time of motor response, in the ventral striatum. In other words: if the neural responses we observed experimentally were the result of spillover from the incentive presentation phase, we would expect to see linearly decreasing parameter fits in our simulated data as a function of incentive level at the time of the motor task.

The results from these simulations can be found in Figure S1A,B.

Analysis of the Physiological Spillover Between Incentive Presentation and the Motor task

To test that the deactivations we observed during the motor task were not the result of a physiological spillover from the incentive presentation, we examined neural responses in trials binned by their jitter between incentive presentation and task performance (i.e., small jitter, [2 s- 3.5 s); large jitter, [3.5 s - 5 s]). The rational being that if deactivations during the motor task were the result of physiological crosstalk from the incentive presentation event, longer jitter duration trials would have less propensity to corrupt neural responses during the motor task, and would result in a trend of diminished effect sizes for trials with longer jitter durations.

To perform this analysis we created a new GLM in which we modeled the incentive presentation event as well as separately modeling motor events which were preceded by short or long jitter durations. Each of these categorical events included a parametric modulator corresponding to the level of incentive offered. We examined parameter estimates at the time of the motor task, extracted from an ROI of 20 mm in radius centered at [x = -0.4, y = 6.1, z = 1.5], encompassing ventral striatum[1]. Observations of these parameter estimates allowed us to determine if the duration of jitter had an influence on neural activity during the motor task condition.

Consistent with the imaging results presented for all trials, neural responses decreased with increasing incentives at the time of the motor task (i.e., negative parameter estimates). Most importantly we did not find a significant difference between parameter estimates for short and long jitter duration trials ($p < 0.42$). These results suggest that the neural deactivations observed during the motor task are not the result of physiological crosstalk from the incentive presentation event.

The results from this analysis can be found in Figure S1C.

Whole Brain Functional Imaging Analysis

In addition to the general linear model (GLM) described in the main text of the paper, we created GLMs which allowed for a whole brain search for regions that potentially underly the behavioral findings. In particular these models focused on identifying the neural activity associated with participants' parabolic performance responses to incentives.

We estimated participant-specific (first-level) general linear models (GLM) that included three types of conditions corresponding to incentive presentation, successful motor task performance, and unsuccessful motor task performance. For the incentive presentation condition we introduced parametric modulators for: (1) first and second order modulation of incentive level; (2) performance (success/failure) on the subsequent motor task; (3) task difficulty (easy/hard); (4) an interaction between first order modulation of incentive level and subsequent task difficulty; (5) an interaction between second order modulation of incentive level and subsequent task difficulty. For the motor task conditions we introduced the following parametric modulators: (1) first and second order modulation of incentive level (2) task difficulty (easy/hard) (3) an interaction between first order modulation of incentive level and task difficulty (4) an interaction between second order modulation of incentive level and task difficulty.

This analysis allowed for a whole brain search for regions that potentially underlie the behavioral finding. We tested several contrasts corresponding to neural activity during incentive presentation and motor task execution. These contrasts tested: 1) areas in which activity had a linear correlation with incentive level; 2) areas in which activity had a parabolic correlation with incentive level; 3) areas that were correlated with an interaction between task difficulty and incentive level; 4) areas that were correlated with an interaction between task difficulty and a parabolic response to incentive; 5) regions that were more/less active between trials that were successful and unsuccessful.

We also created a GLM that, instead of modeling a perfectly quadratic response to incentive, used actual participant performance, in addition to the same regressors mentioned above.

For both of these GLMs the only contrasts that showed significant activations ($p<0.001$) during whole brain analysis were positively correlated with incentive level during incentive presentation, and negatively correlated with incentive level during the motor task.

The results from this analysis can be found in Figure S1D.

fMRI Region of Interest Analysis

All results reported in the main text are with a corrected significance threshold of $P<0.05$, based on small-volume correction within an anatomically defined region of interest (ROI), bilaterally encompassing the ventral striatum (incorporating the nucleus accumbens extending into the ventral parts of the putamen; Supplemental Figure S1D).

All effect size and percent signal change plots for the incentive presentation and motor task contrasts are reported using the average voxel activity in a sphere 20 mm in radius, centered at [x = -0.4, y = 6.1, z = 1.5]. These ventral striatal coordinates were chosen because Tom et al. found this region to commonly encode potential gains [1].

The percent signal change graphs were generated from the GLM described above. To extract percent signal changes for the parametric modulator at the time of incentive presentation we decomposed the modulator into bins for each level of incentive. New onset regressors were then recreated that contained all events in a particular incentive bin. The beta weights for these new regressors were re-estimated and percent signal changes were calculated for the bins. Percent signal changes for the motor task were simply calculated for each modeled condition separately.

Neural Sensitivity to Incentive

Neural sensitivity measures were generated for individual participants both during the incentive presentation and the motor task frames. The sensitivity measure was defined as the slope of a linear relationship between the incentive levels and its corresponding percent signal change extracted from a sphere 20 mm in radius, centered at [x = -0.4, y = 6.1, z = 1.5] (as described above).

Kinematic Trajectory Analysis

To ensure participants' movement kinematics were the same across incentive levels and that our imaging results were not confounded by differing motor output for incentive, we calculated measures of hand kinematics during task performance.

Hand Accuracy: Endpoint accuracy was calculated as the distance between a participant's final hand position and the target center (Euclidean norm).

Mean Velocity: This velocity metric was the mean hand velocity over the course of a movement trajectory.

Hand Smoothness: This metric captures the average rate of change of the acceleration of movement (jerk). It is calculated by dividing the negative mean jerk magnitude by the peak speed. Taking the negative of the mean jerk causes increases in the jerk metric to correspond with increases in smoothness; in this way it transforms the jerk metric from a measure of "nonsmoothness" into a measure of smoothness. Normalizing the mean jerk by the peak speed makes the measure robust to confounds arising from changes in overall movement speed. This method was introduced in Roher et al. [10].

The results from these analyses can be found in Figure S2.

Independent Replication of Neural Sensitivity / Loss Aversion Correlation

We performed an additional fMRI experiment to confirm the relationship between neural deactivation during motor performance and behavioral loss aversion. This study exactly duplicated the hard difficulty condition of the initial fMRI study, in addition to including additional experimental conditions. We have confined the data and analysis presented here to only those trials that duplicate our initial experimental conditions. The results from the additional conditions are beyond the scope of this manuscript and we are in the processes of analyzing them for later publication.

For this new experiment participants first performed the training and thresholding phases, as in the experiment presented in the main text. They then returned on the following day and performed the task for incentive while being scanned with fMRI. During the experiment participants performed trials exactly as described in the main text. The target size was sized to produce an unincentivized rate of success of 60% (the hard difficulty condition). Trials were for potential gains ranging from $0 to $100 in increments of $25. Each incentive condition consisted of 30 trials. At the end of the experiment a single trial was extracted and participants were paid based on performance on that trial. Following the testing phase we obtained a measure of participants behavioral loss aversion and risk aversion using the choice tasks described in the main text.

The fMRI data was analyzed in a similar fashion as described in the main text. We included separate categorical regressors corresponding to the onset of incentive presentation, as well separate categorical regressors for the onset of the motor task for successful and unsuccessful trials. Parametric modulators corresponding to the presented incentive were included for each of these categorical regressors.

We confined the presentation of our results to successful trials. Neural sensitivity measures were extracted from average voxel activity in a sphere 20 mm in radius, centered at [x = 21, y = 9, z = -9]. These coordinates were chosen because they were found in our initial study to encode a deactivation correlated with behavioral loss aversion. As shown in our first study, the extent of participants' neural deactivation during the motor task was correlated to a measure of their behavioral loss aversion

The results from this experiment can be found in Figure S3.

Supplemental References

1. Tom, S.M., Fox, C.R., Trepel, C. & Poldrack, R.A. The Neural Basis of Loss Aversion in Decision-Making Under Risk. *Science* **315**, 515--518 (2007).
2. O'Doherty, J.P. Reward representations and reward-related learning in the human brain: insights from neuroimaging. *Current Opinion in Neurobiology* **14**, 769-776 (2004).
3. Knutson, B., Taylor, J., Kaufman, M., Peterson, R. & Glover, G. Distributed Neural Represenations of Expected Value. *Journal of Neuroscience* **25**, 4806-4812 (2005).
4. Rushworth, M.F.S., Krams, M. & Passingham, R.E. The Attentional Role of the Left Parietal Cortex: The Distinct Lateralization and Localization of Motor Attention in the Human Brain. *Journal of Cognitive Neuroscience* **13**, 698-710 (2001).
5. Coull, J.T. Neural correlates of attention and arousal: insights from electrophysiology, functional neuroimaging and psychopharmacology. *Progress in Neurobiology* **55**, 343-361 (1997).
6. Critchley, H.D., Melmedc, R.N., Featherstonea, E., Mathias, C.J. & Dolan, R.J. Volitional Control of Autonomic Arousal: A Functional Magnetic Resonance Study. *Neuroimage* **16**, 909-919 (2002).
7. Dingwell, J.B., Mah, C.D. & Mussa-Ivaldi, F.A. Manipulating Objects With Internal Degress of Freedom: Evidence for Model-Based Control. *Journal of Neurophysiology* **88**, 222-235 (2002).
8. Astrom, K.J. & Murray, R.M. *Feedback Systems: An Introduction for Scientists and Engineers* (Princeton University Press, 2008).
9. Handwerker, D.A., Ollinger, J.M. & D'Esposito, M. Variation of BOLD hemodynamic responses across subjects and brain regions and their effects on statistical analysis. *Neuroimage* **21**, 1639-1651 (2004).
10. Roher, B., *et al.* Movement Smoothness Changes during Stroke Recovery. *Journal of Neuroscience* **22**, 8297-8304 (2002).
